# Risk Factors of Bacteremia following Multiple Traumas

**DOI:** 10.1155/2020/9217949

**Published:** 2020-04-06

**Authors:** Hak-Jae Lee, Eol Choi, Nak-Joon Choi, Hyun-Woo Sun, Jae-Suk Lee, Jeong-Woo Lee, Tae-Yoon Kim, Yoon-Joong Jung, Suk-Kyung Hong

**Affiliations:** ^1^Division of Acute Care Surgery, Department of Surgery, University of Ulsan College of Medicine, Asan Medical Center, 88 Olympic-ro 43-gil, Songpa-gu, Seoul 05505, Republic of Korea; ^2^Division of Vascular Surgery, Department of Surgery, University of Ulsan College of Medicine, Asan Medical Center, 88 Olympic-ro 43-gil, Songpa-gu, Seoul 05505, Republic of Korea

## Abstract

**Background:**

Bacteremia is a major nosocomial infection that frequently occurs in trauma patients, increasing morbidity and mortality. The aim of this study was to identify risk factors and to describe epidemiological patterns for early onset (EOB) and late onset (LOB) bacteremia after trauma.

**Methods:**

We retrospectively reviewed medical records of all trauma patients admitted to the surgical intensive care unit and general ward between January 2011 and December 2015. The information was collected for each patient and recorded in a computer database: early onset bacteremia (EOB) was defined as when onset occurred within 7 days after trauma, and late onset bacteremia (LOB) was defined as when onset occurred after 7 days from trauma.

**Results:**

Thirty-four patients of 859 (4%) developed bacteremia during their hospital stay: 4 (11.8%) developed EOB, 26 (76.4%) LOB, and 4 (11.8%) patients developed both of them. Sixty events of bacteremia happened to these patients: 9 (15.0%) EOB and 51 (85.0%) LOB. Gram-positive cocci were isolated more frequently than Gram-negative bacilli in both groups. Gram-positive cocci were more frequently isolated in EOB than in LOB; otherwise, there was no statistical significance (77.8% vs. 64.7%, *p*=0.683). Central line-associated blood stream infection (CLABSI) and surgical site infection (SSI) were the most common identified source for LOB. Presence of liver (OR: 2.66, *p*=0.035) and pelvic injury (OR: 2.25, *p*=0.038), gastrointestinal tract perforation (OR: 5.48, *p*=0.002), and massive transfusion (OR: 3.36, *p*=0.006) represented risk factors for bacteremia.

**Conclusions:**

Presence of pelvic and liver injury on arrival in emergency department, gastrointestinal tract perforation, and massive transfusion within the first 24 hours after trauma appears to be significant risk factors for bacteremia.

## 1. Introduction

Bacteremia is a significant cause of morbidity and mortality in critically ill patients [1]. In particular, patients with severe trauma are at greater risk of bacteremia due to the destruction of the skin barrier and the invasive procedures performed on these patients [2, 3]. Blood stream infections (BSI) increase the length of stay, risk of in-hospital death, and hospital costs of trauma patients [4, 5].

The factors affecting the risk of bacterial infections in trauma patients include damage to the natural barriers such as skin; long-term application of invasive devices, such as central venous catheter or endotracheal tube due to intensive care; surgical treatment such as debridement and drainage of injured tissue; and degradation of the immune system due to massive blood loss and transfusion [2, 6].

Bacteremia may reflect a failure of the host's innate immune response due to a series of immunological responses to severe injury resulting in immunoparalysis [7]. Posttraumatic complications are associated with overproduction of proinflammatory mediators and imbalances of cell-regulated innate immunity. The persistent imbalance of immune system can lead to hyperinflammation or immunosuppression, ultimately resulting in multiple organ dysfunction [7, 8]. Patients that present with systemic inflammatory response of nonbacterial etiology at initial admission of hospital often develop sepsis as a consequence of bacterial superinfection, and long-term immunosuppression after traumatic injury could increase the frequency of opportunistic infections [9]. Sepsis that occurs during the period of trauma-induced immune dysfunction is associated with higher mortality, and therefore more aggressive treatment is needed [8, 10, 11].

Analysis of the epidemiological and clinical aspects of bacteremia is important for developing treatment strategies. Antonelli et al. suggested that the early-onset bacteremia (<96 hours) was more likely to occur after abdominal and thoracic injuries, while the risk factors for late-onset bacteremia (≥96 hours) included intravascular catheterization and mechanical ventilation [2, 4]. Recognition of potential bacterial pathogens causing the bacteremia in trauma patients may also be helpful in the use of prophylactic and empirical antimicrobial therapy [12].

The aim of this study was to describe epidemiological patterns for early-onset (EOB) and late-onset (LOB) bacteremia and to identify risk factors for bacteremia after trauma.

## 2. Methods

### 2.1. Patients and Data Collection

Patients admitted to Asan Medical Center with trauma from January 2011 to December 2015 were enrolled in this study. A total of 859 patients were admitted to the intensive care unit (ICU) or general ward through the emergency department during this period. The following information was collected for each patient: demographics; severity of trauma according to injury severity score (ISS) and abbreviated injury score (AIS); severity of coma according to the Glasgow Coma Scale (GCS); presence of pneumothorax, chest contusion, hemothorax, liver injury, gastrointestinal tract perforation, pancreas injury, soft tissue injury, and pelvic injury; use of mechanical ventilation; and presence of shock and transfusion at admission to the emergency department. All medical records and radiographic images were reviewed retrospectively. The study was approved by the institutional review board of Asan Medical Center (institutional review board no. 2018-1583). This trial is registered with NCT04042636.

### 2.2. Definitions

An episode of bacteremia was defined as the first or new positive blood culture obtained more than 48 hours after the preceding positive blood culture. Blood cultures were performed when the body temperature was above 38 degrees or higher or when the infection was suspected. Early-onset bacteremia (EOB) was defined as bacteremia occurring within 7 days after trauma. All bacteremia appearing after 7 days from trauma defined as late onset bacteremia (LOB). Blood samples were collected from peripheral blood vessels and arterial/venous catheters and were sorted by site of blood sampling. All samples were cultured in Bactec culture bottles and incubated for 5 days. Antimicrobial susceptibility was performed by the disc diffusion method [13]. The antibiotic therapy was considered appropriate if at least one effective drug was included in the empiric and in the definitive antibiotic treatment [14].

Shock was defined as systolic blood pressure less than 90 mmHg or a mean arterial pressure less than 60 mmHg or reduced by more than 30 mmHg in a hypertensive patients for at least 30 minutes [15].

Central line-associated blood stream infection (CLABSI) was defined as a primary BSI in a patient that had a central line within the 48 hours period before the development of a BSI and that was not related to an infection at another site. The tip of central vascular catheters, if received, was processed by the roll plate method developed by Maki et al. [16]. Surgical site infections (SSIs) were infections of the incision or organ or space that occurred after surgery [17]. The diagnosis of surgical site infection was based on tissue culture, purulent discharge on devitalized tissue, or cultures for drainage from wound. Urinary tract infection (UTI) was defined as the presence of bacteria in the catheter urine of at least 10^5^ colony-forming units (CFU)/ml with clinical signs or symptoms [18]. Ventilator-associated pneumonia (VAP) was defined as pneumonia that occurred 48 hours after mechanical ventilation, characterized by clinical sign of systemic infection (e.g., fever and elevated white blood cell count), the presence of purulent sputum, progressive infiltrate, or a positive microbiological culture [19].

### 2.3. Statistical Analysis

We used Student's *t*-test for comparing continuous variables. The chi-squared test or Fisher's exact test was used to compare the categorical variables to assess the risk factors for bacteremia. Logistic stepwise regression analysis was performed to predict the risk factors for bacteremia based on clinical variables. The significance level was set at *p* < 0.05. Adjusted odds ratios and 95% confidence intervals were derived. Statistical analyses were conducted with the R program (version 3.3.2; R Foundation for Statistical Computing, Vienna, Austria; http://www.R-project.org).

## 3. Results

Of 859 patients admitted with trauma, 71.6% were male, and the mean age of patients was 48.8 years. At the time of admission, the mean ISS was 20.6 ± 16.5 and GCS score was 13.3 ± 3.6. 161 (18.7%) patients were in a state of shock at the time of admission. 254 (29.6%) patients received transfusion, of which 70 (8.1%) patients received massive transfusions of more than 10 units of packed red blood cells (pRBCs). A total of 459 (53.4%) patients were admitted the intensive care unit (ICU). A total of 783 blood cultures were performed, 60 (7.7%) of which were positive for bacteremia. Bacteremia occurred in 34 (4.0%) patients during their hospital stay: 4 (11.8%) patients developed EOB, 26 (76.4%) patients developed LOB, and 4 (11.8%) patients developed both EOB and LOB. In 859 patients with trauma, 64 (7.5%) patients died (Table 1).

We compared the clinical variables between patients with bacteremia and those who did not (Table 2). The clinical variables used were compared with data such as transfusion, shock, and emergency operation to evaluate severity status that could affect bacteremia. There were no significant differences in age, gender, and cause of injury. ISS tended to be higher in the bacteremia group (*p*=0.061). When comparing each AIS, abdominal (1.3 ± 1.5 vs. 2.4 ± 1.5, *p* < 0.001) and extremity AIS (1.5 ± 1.5 vs. 2.1 ± 1.6, *p*=0.035) were statistically higher in the patients with bacteremia compared with patients without bacteremia. The presence of shock (17.9% vs. 38.2%, *p*=0.006) and blood transfusion (28.0% vs. 50.0%, *p*=0.010) at the time of admission were also more frequent in the patients with bacteremia compared with those without bacteremia. Massive transfusions with more than 10 units of pRBCs were performed four times in patients with bacteremia than those who did not develop bacteremia (7.3% vs. 29.4%, *p* < 0.001). Emergency operation included laparotomy and orthopedic surgery performed two times in patients with bacteremia (33.7% vs. 62.2%, *p* < 0.001).

Table 3 shows the distribution of micro-organisms isolated in patients with EOB versus patients with LOB. Overall, 60 events of bacteremia occurred in a total of 34 patients with bacteremia. Gram-positive cocci were isolated more frequently than Gram-negative bacilli in both groups. Gram-positive cocci were more frequently isolated in EOB than in LOB: otherwise, there was no statistical significance (77.8% vs. 64.7%, *p*=0.683). Of the 60 events of bacteremia, 4 cases (6.7%) were polymicrobial. Two of the 9 bacterial isolates in EOB were Gram-negative, *Acinetobacter* spp. and *Escherichia coli*. The proportion of Gram-negative bacilli and *Candida* spp. increased with late onset period. The number of multidrug-resistant organism was significantly higher in the LOB period compared with the EOB period (78.4% vs. 33.3%, *p*=0.018).

We also analyzed the cause of infection in EOB and LOB (Table 4). Of the 8 cases of early onset bacteremia, there was only 1 case of CLABSI, and the remaining 7 cases were SSIs caused by soft tissue or abdominal injuries. CLABSI accounted for 52.1% of LOB cases. SSIs were the second most common cause of infection, seen in 15 cases (31.2%), but its frequency decreased. In the LOB period, other causes of infection including pneumonia, urinary tract infection (UTI), and meningitis that were not seen in EOB cases also appeared.

Potential risk factors for bacteremia were analyzed (Supplementary Table 1). As ISSs greater than 17 were significantly associated with incidence of bacteremia (*p*=0.003), among six categories of AIS, abdominal (*p*=0.001) and extremity AISs (*p*=0.028) were significantly associated with bacteremia. In addition, the presence of shock (OR: 2.83, 95% CI: 1.35–5.76, *p*=0.006) and massive transfusion (OR: 5.34, 95% CI: 2.32–11.45, *p* < 0.001) at the time of admission were significantly associated with the likelihood of bacteremia.

Based on the above results, we analyzed the predictors of bacteremia by using multivariable logistic regression with age and gender adjustments (Table 5). ISS, GCS, injury mechanisms of various organs, shock, and massive transfusion were analyzed as variables. The factors—liver injury, gastrointestinal tract perforation, pelvic injury, and massive transfusion—were statistically significant predictors of bacteremia.

## 4. Discussion

In the past decade, there have been many studies on bacteremia in trauma patients. In particular, bacteremia had been reported to have a greater impact on clinical outcomes due to change in the immune system of patients with trauma or surgery [2, 4, 8, 10, 20]. In patients with bacteremia, more intensive care is needed because of the increased length of stay in ICU and mortality [4, 21, 22]. In our study, clinical outcomes of 459 patients admitted to the ICU were compared between the bacteremia group (*n* = 32) and nonbacteremia (*n* = 427) group. Although there was no significant difference in mortality, there was a difference in the length of ICU stay (8.1 ± 13.7 vs. 26.5 ± 31.7, *p*=0.003) and the days of mechanical ventilator use (5.9 ± 13.3 vs. 24.5 ± 32.2, *p*=0.003).

There have been many discussions about the use of prophylactic antibiotics in trauma patients [23–26]. However, improper use of antibiotics in trauma patients may increase the antibiotic resistance. Recently, the incidence of multidrug-resistant organism infections has been increasing in various trauma ICUs [27–29]. Indeed, in our study, the incidence of bacteremia was not as high as 7.7%. This is because the most common cause of fever in trauma patients was systemic inflammatory response syndrome. Therefore, it is necessary to understand the causes of infection and the patterns of possible pathogens and to use appropriate antibiotics after trauma.

As seen in other studies, the main cause of bacteremia in our study was Gram-positive bacteremia (GPB) [2, 30–32]. GPB was accounted for 77.8% of EOB and 68.4% of LOB infections. However, although not statistically significant, the frequency of Gram-negative bacteremia (GNB) was higher in LOB (22.2% ⟶ 31.4%). This is similar to findings of other studies that analyzed the trend of early and late onset bacteremia. Antonelli et al. reported GPB was 78.6% of EOB (defined as happening within 4 days after trauma), while GNB was more frequent as 49.1% in LOB [2]. Lee et al. analyzed EOB (defined as happening within 2 months after surgery) and LOB (beyond 2 months) in liver transplant patients; although there were differences in the period of time, the frequency of GPB was higher in EOB, while the frequency of GNB was higher in LOB (GPB: 58.7% ⟶ 41.3%, GNB: 36.2% ⟶ 63.8%, *p*=0.01) [31]. Therefore, it is more important to pay attention to the antimicrobial coverage of GNB in LOB.

The strength of our study is that it evaluated risk factors that can affect bacteremia at the time of admission. Patients with abdominal or pelvic injury, who received massive transfusions, or who developed shock were more likely to develop bacteremia. Several other studies have also reported that shock and transfusion were associated with the incidence of infection. Rello et al. suggested that shock was the only risk factor of bacteremia in ICU patients [1]. Robert et al. reported that infection rates (OR: 1.5) and mortality rates (24.3% vs. 10.2%) were significantly higher in transfusion recipients. Sadjadi et al. found that the risk of infection was more than eight times higher in patients who received transfusions than those who did not and that the incidence of pneumonia, line infection, and abscess were higher in the patients who received more than 10 units of packed red blood cells (pRBCs) [6]. This is the reason that pRBCs act as immunosuppressants. Therefore, a more aggressive antibiotic therapy is needed in severe trauma patients, who had higher ISS, shock, or received a massive transfusion at the time of admission.

Our study also found that injury to intra-abdominal organs, such as liver, pancreas, gastrointestinal tract, and the pelvic region are the main causes of bacteremia. In the case of intraperitoneal injury, bacteremia may occur due to intraperitoneal propagation of intestinal bacteria. Antonelli et al. reported that, in addition to abdominal injury, thoracic trauma also was associated with the occurrence of EOB [2]. Thoracic injury mainly causes development of pneumonia, but it is difficult to develop early onset bacteremia. In our study, thoracic injury was not associated with bacteremia. Edelman et al. reported that stomach, pancreas, and colon injuries, transfusion, and shock were independent risk factors for developing bacteremia [33]. Stoutenbeek et al. argued that selective decontamination of the digestive tract is necessary in patients with multiple injuries [32]. Song et al. reported that pelvic infection progressed in 50% of patients with open pelvic fracture [34]. Large blood vessels are distributed in the pelvis, leading to massive bleeding at the time of injury. In addition, it is easy to accompany damage to the surrounding small intestine and rectum, increasing the likelihood of infection.

There were several limitations to this study. First, the frequency and number of patients with bacteremia compared with the entire trauma patients are low, which can lead to bias as even small variations can result in a large change in the overall frequency in patients with bacteremia. Second, our study was a retrospective analysis with the inherent drawbacks on data available for analysis. Furthermore, because there are multiple interrelated factors that lead to bacteremia in unstable trauma patients, the incidence of bacteremia is high and the clinical outcome is poor.

## 5. Conclusion

In patients with multiple trauma, changes in the immune system can increase susceptibility to bacteremia, which leads to poor clinical outcomes. Therefore, it is necessary to accurately analyze the possible causes of infection and pathogens according to the time of injury and to administer appropriate antibiotics. If there is shock at the time of admission or if a massive transfusion is required, aggressive antibiotics treatment should be considered. In addition, the incidence of bacteremia should also be considered for abdominal and pelvic region injuries. In the early stages of trauma treatment, antibacterial coverage of GPB should be applied and the coverage of GNB should also be considered over time.

## Figures and Tables

**Table 1:** Characteristics of the study population.

Characteristics	*N* (%) or mean ± SD (*n* = 859)
Age (years)	48.8 ± 18.2
Gender (male, %)	615 (71.6%)
Cause of injury	
Pedestrian TA	147 (17.1%)
In car TA	228 (26.5%)
Motorcycle TA	123 (14.3%)
Fall down	219 (25.5%)
Others	142 (16.5%)
ISS	20.6 ± 16.5
GCS	13.3 ± 3.6
Shock (*n*, %)	161 (18.7%)
Transfusion (*n*, %)	254 (29.6%)
Massive transfusion (more than 10 pRBCs) (*n*, %)	70 (8.1%)
Admitted ICU (*n*, %)	459 (53.4%)
Bacteremia (*n*, %)	34 (4.0%)

TA, traffic accident; GCS, Glasgow coma scale; ISS, injury severity score; pRBC, packed red blood cell; ICU, intensive care unit.

**Table 2:** Comparisons of characteristics between the bacteremia group and nonbacteremia group.

	Nonbacteremia (*n* = 825)	Bacteremia (*n* = 34)	*p* value
Age (years)	48.7 ± 18.1	51.1 ± 18.7	0.442
Sex (male, %)	235 (28.5%)	9 (26.5%)	0.951
Cause of injury			0.503
Pedestrian TA	143 (17.3%)	4 (11.8%)	
In car TA	218 (26.4%)	10 (29.4%)	
Motorcycle TA	115 (13.9%)	8 (23.5%)	
Fall down	211 (25.6%)	8 (23.5%)	
Others	138 (16.7%)	4 (11.8%)	
ISS	20.4 ± 16.5	25.8 ± 14.9	**0.061**
AIS			
Head AIS	1.0 ± 1.7	0.9 ± 1.3	0.636
Face AIS	0.6 ± 1.1	0.9 ± 1.5	0.247
Chest AIS	1.6 ± 1.6	1.7 ± 1.6	0.729
Abdominal AIS	1.3 ± 1.5	2.4 ± 1.5	^*∗*^ **0.001**
Extremity AIS	1.5 ± 1.5	2.1 ± 1.6	^*∗*^ **0.035**
External AIS	0.3 ± 0.8	0.5 ± 0.9	0.189
GCS	13.4 ± 3.6	13.0 ± 3.6	0.573
Shock (*n*, %)	148 (17.9%)	13 (38.2%)	^*∗*^ **0.006**
Emergency operation (n, %)	277 (33.7%)	23 (62.2%)	^*∗*^ **0.001**
Transfusion (*n*, %)	231 (28.0%)	17 (50.0%)	^*∗*^ **0.010**
Massive transfusion (more than 10 pRBCs) (*n*, %)	60 (7.3%)	10 (29.4%)	^*∗*^ **0.001**

TA, traffic accident; GCS, Glasgow coma scale; ISS, injury severity score; pRBC, packed red blood cell; AIS, abbreviated injury scale. ^*∗*^Significance set as *p*=0.05.

**Table 3:** Etiology of early- and late-onset bacteremia.

	Early-onset bacteremia (*n* = 9)	Late-onset bacteremia (*n* = 51)	*p* value
Gram (+) cocci (*n*, %)	7 (77.8%)	33 (64.7%)	0.683
*Staphylococcus aureus*	3	8	
*Streptococcus epidermidis*	2	6	
Other CoNS	1	12	
*Enterococcus* spp.	1	6	
*Corynebacterium* spp.	0	1	
Gram (-) bacilli (*n* %)	2 (22.2%)	16 (31.4%)	
*Acinetobacter* spp.	1	9	
*Enterobacter* spp.	0	1	
*Escherichia coli*	1	1	
*Klebsiella pneumoniae*	0	3	
*Serratia* spp.	0	1	
*Stenotrophomonas* spp.	0	1	
*Candida* spp. (*n*, %)	0	2 (3.9%)	
The number of multidrug-resistant organisms (*n*, %)	3 (33.3%)	40 (78.4%)	^*∗*^ **0.018**

CoNS, coagulase-negative staphylococci; spp., species.

**Table 4:** Infection source of early and late onset bacteremia.

	Early-onset bacteremia (*n* = 8)	Late-onset bacteremia (*n* = 48)
CLABSI	1 (12.5%)	25 (52.1%)
SSI	7 (87.5%)	15 (31.2%)
Unknown	0	1 (2.1%)
UTI	0	2 (4.2%)
CNS infection	0	3 (6.3%)
Pneumonia	0	2 (4.2%)

CLABSI, central line-associated blood stream infection; SSI, surgical site infection; UTI, urinary tract infection; CNS, central nervous system.

**Table 5:** Multivariable logistic regression for risk factors of bacteremia (*n* = 34).

	Adjusted OR (95% CI)	*p* value
Liver injury	**2.66 (1.01–6.33)**	**0.035**
Gastrointestinal tract perforation	**5.48 (2.11–13.15)**	**0.002**
Pelvic injury	**2.25 (1.03–4.82)**	**0.038**
Massive transfusion	**3.36 (1.35–7.74)**	**0.006**

## Data Availability

The datasets used and/or analyzed during the current study are available from the corresponding author on reasonable request.

## Abbreviations

EOB: Early-onset bacteremia
LOB: Late-onset bacteremia
ISS: Injury severity score
AIS: Abbreviated injury score
GCS: Glasgow Coma Scale
CLABSI: Central line-associated blood stream infection
SSI: Surgical site infection
OR: Odds ratio
BSI: Blood stream infection
ICU: Intensive care unit
UTI: Urinary tract infection
VAP: Ventilator associated pneumonia
TA: Traffic accident
CFU: Colony-forming unit
SD: Standard deviations
CI: Confidence interval
pRBC: Packed red blood cell
CNS: Central nervous system
GPB: Gram-positive bacteremia
GNB: Gram-negative bacteremia.

## Supplementary Materials

Supplementary Materials Supplementary Table 1: risk factors for bacteremia (*n* = 34).
