# Supplementary material for: Risk Factors of Bacteremia following Multiple Traumas
Source: Emerg Med Int. 2020 Apr 6;2020:9217949. doi: 10.1155/2020/9217949 (PMC7165339; doi:10.1155/2020/9217949)
Supplement: Supplementary Materials — Supplementary Table 1: risk factors for bacteremia (n = 34). [file 9217949.f1.pdf]

Supplementary Table 1: Risk factors for bacteremia (n = 34)

	No. of patients (n)	No. of bacteremia patients (n, %)	Adjusted OR (95% CI)	*p*-value
**ISS**				***0.003**
**≤ 16**	**416**	**7 (1.9%)**		
**17 >**	**443**	**27 (6.1%)**		
**Head AIS**				**0.705**
**0**	**572**	**22 (3.8%)**		
**1-3**	**210**	**11 (5.2%)**		
**4-6**	**77**	**1 (1.3%)**		
**Face AIS**				**0.125**
**0**	**630**	**23 (3.7%)**		
**1-3**	**213**	**8 (3.8%)**		
**4-6**	**16**	**3 (19.7%)**		
**Chest AIS**				**0.378**
**0**	**392**	**15 (3.8%)**		
**1-3**	**406**	**14 (3.4%)**		
**4-6**	**61**	**5 (8.2%)**		
**Abdominal AIS**				***0.001**
**0**	**444**	**8 (1.8%)**		
**1-3**	**322**	**17 (5.3%)**		
**4-6**	**93**	**9 (9.7%)**		
**Extremity AIS**				***0.028**
**0**	**403**	**11 (2.7%)**		
**1-3**	**382**	**17 (4.5%)**		
**4-6**	**74**	**6 (8.1%)**		
**External AIS**				**0.086**
**0**	**718**	**24 (3.3%)**		
**1-3**	**131**	**10 (7.6%)**		
**4-6**	**10**	**0**		
**Rib fracture**	**335**	**17 (5.1%)**		**0.245**
**Hemothorax**	**111**	**8 (7.2%)**		**0.105**
**Liver injury**	**92**	**8 (8.7%)**	**2.709 (1.027-6.415)**	***0.022**
**Pancreas injury**	**19**	**3 (15.8%)**	**4.874 (0.869-18.362)**	***0.036**
**Bowel perforation**	**57**	**9 (15.8%)**	**5.804 (2.256-13.759)**	***0.001**
**Pelvic injury**	**217**	**14 (6.5%)**	**2.143 (0.982-4.554)**	***0.047**
**Degloving injury**	**22**	**1 (4.5%)**		**0.593**
**Urogenital injury**	**27**	**2 (7.4%)**		**0.290**
**Shock**	**161**	**13 (8.1%)**	**2.827 (1.349-5.764)**	***0.006**
**Massive Transfusion**	**70**	**10 (14.3%)**	**5.337 (2.323-11.452)**	***0.001**

ISS, Injury severity score; AIS, abbreviated injury scale.

* Significance set as $p = 0.05$

18
